# Migraine start, course and features over the cycle of combined hormonal contraceptive users with menstrual migraine – temporal relation to bleeding and hormone withdrawal: a prospective diary-based study

**DOI:** 10.1186/s10194-020-01150-1

**Published:** 2020-06-24

**Authors:** Gabriele S. Merki-Feld, Nina Caveng, Gina Speiermann, E. Anne MacGregor

**Affiliations:** 1 Department of Reproductive Endocrinology, University Hospital Zürich, Frauenklinikstrasse 10, CH - 8091 Zürich, Switzerland; 2 Centre for Reproductive Medicine, St Bartholomew’s Hospital, London, UK

**Keywords:** Hormone, Estrogen withdrawal, Menstrual migraine, Menstrual bleeding, Contraception, Triptans, Combined pill, Migraine prevention

## Abstract

**Background:**

Many studies have described the features of menstrually-related migraines (MRM) in the natural cycle and the efficacy of prevention. MRM in combined hormonal contraceptive (CHC) users has scarcely been researched. Estrogen and progestin withdrawal in CHC users are both more abrupt and from higher hormone levels compared with the natural cycle. An advantage for prevention of MRM in CHC users is that the hormone withdrawal is predictable. It is unknown, whether the attacks during the hormone-free interval are associated with the hormone withdrawal or onset of bleeding. Improved understanding of this relation might contribute to better define and shorten the time interval for prevention.

**Methods:**

For this prospective diary-based trial we collected migraine and bleeding data from CHC users with MRM in at least two of three cycles. We analyzed frequency of migraines over the whole CHC cycle. During the hormone-free phase the relation between onset of migraine and onset of bleeding was studied. We compared pain intensity and identified prolonged-migraine attacks during hormone use and the hormone-free phase.

**Results:**

During the hormone-free interval the number of migraine days and the pain score/migraine day were significantly higher in comparison with the mean during hormone use. The prevalence of migraine attacks was fourfold on hormone-free days 3–6. Migraine typically started on days 1–4. Migraine in relation to bleeding mostly occurred on days − 1 to + 4. In 78% of the cycles the first migraine day occurred during bleeding days 1 ± 2 and 48% started on days − 1 and day 1. The predictability of the first bleeding day was very high.

**Conclusion:**

The day of hormone-withdrawal migraine and the first bleeding day are highly predictable in CHC users. Migraine onset is mostly day − 1 and 1 of the bleeding and on days 1–4 of the hormone-free interval. Migraine attacks of CHC users in the hormone-free interval are severe and long lasting. Further trials are necessary to investigate if this knowledge can be used to optimise prevention.

## Background

Migraine affects approximately 18% of women and is ranked as the third most prevalent and seventh leading cause of disability [1, 2]. Both incidence and prevalence of migraine are 2–3 fold higher in women than in men during the reproductive years and more than 50% of women report an association between migraine and menstruation [3, 4]. The International Classification of Headache Disorders (ICHD-3) defines pure menstrual migraine (PMM) as migraine occurring only on days 1 ± 2 of menstruation in at least two of three cycles and menstrually-related migraine (MRM) if migraine also occurs on other days of the cycle [5]. Estrogen withdrawal is a recognised trigger for menstrual attacks in the natural cycle and in women using combined hormonal contraceptives (CHC) [6–8]. Estrogen-withdrawal headache (EWH) is defined as a headache or migraine, which develops within 5 days after daily consumption of exogenous estrogen for three weeks or longer, which has been interrupted. Prostaglandins released from the endometrium during the phase of menstrual bleeding might also play a key role in the pathophysiology of menstrual attacks [9]. MRM attacks arecharacterized as more severe, of longer duration and more resistant to treatment compared to non-menstrual attacks [10–14]. With limited options for successful acute treatment, the high burden and the high prevalence of MRM demonstrate the need for a better understanding of pathophysiology involved. Many studies have described the features of PMM and MRM in natural cycles and the efficacy of acute therapy or short-term prevention over 5–7 days around the menstrual bleeding [10, 12, 14–21]. In contrast, the course, predictability and features of PMM and MRM in CHC users have scarcely been researched, despite oral contraception being used by more than 20% of women of reproductive age in European countries [7, 22–24]. A better understanding might allow more successful individually tailored treatment of these headaches.

Estrogen and progestin withdrawal in CHC users are more abrupt and follow higher estrogen levels compared with withdrawal in the natural cycle. Currently, it is still unknown if migraine during the hormone free interval (HFI) in CHC users is associated with estrogen withdrawal or with prostaglandin release associated with the withdrawal bleed, or both. This is important for considering both type and timing of prevention, especially if symptomatic treatment of menstrual attacks in CHC users with MRM shows the same low response as it is known from MRM in the natural cycle. The aim of this prospectively conducted diary-based study was to identify the course and features of migraines in CHC users diagnosed with menstrual migraine (MRM or PMM) and to understand the temporal relationship between onset of migraine in the HFI and the first day of bleeding. We further analysed within woman variation in the day of migraine onset and bleeding onset in the HFI over several cycles.

## Methods

This prospective trial was conducted at the Clinic for Reproductive Endocrinology at the University Hospital of Zurich, where one of the authors (GSM) runs a clinic for hormonal migraine. Data were collected from November 2017 to May 2019. Participants were recruited at the clinic and through advertisement. We contacted interested women by phone to explain the study and clarify questions. We choose an observation period of 3 cycles, as ICHD-3 recommends use of prospectively conducted diaries over this period for research purposes [5]. We checked whether their headaches during the HFI were migraine and if they complied with the criteria for inclusion. Women were included if they used CHC (oral tablets, transdermal patches, or vaginal ring) in the standard regimen of 21 days with a 7-day HFI, reported migraine regularly occurring during the HFI in at least two out of three cycles, experienced withdrawal bleeding, planned to continue the use of their CHC for at least 3 more cycles, agreed to conduct daily diaries for 3 cycles and gave written informed consent. Exclusion criteria comprised CHC use in flexible or extended cycles, no CHC use, CHC use with shorter HFIs such as 24/4, and migraine during CHC use but not during the HFI. After written consent was obtained, we collected demographic data and a more detailed migraine history during a semi-structured interview. Women who continued to fulfil the inclusion criteria were asked to complete diaries for 3 cycles of a 21/7 CHC regimen (84 days). The diaries collected daily information on occurrence, intensity and duration of migraine attacks, use of pain medication, CHC use and uterine bleeding. Pain intensity was rated in the diaries according to a 4 point scale (0 = no pain; 3 = severe pain). This score is easy to understand and has been proven to be useful in daily conducted diaries of migraineurs. As for the natural cycle and in previous studies in CHC users it has been found, that the long duration of MRM is a big burden, we identified in addition to migraine days those migraine attacks with a duration of more than 24 h. Diaries of participants were excluded from the final data analyses, if migraine was not reported in at least two of three cycles. Furthermore, diaries from women who stopped CHC use, withdraw consent or became pregnant were excluded. The study has been approved by the regional ethics committee (KEK-stV-Nr 2016–01791) and was registered on clinicaltrials.gov: NCT04012593.

### Statistical analyses

The programmes IBM SPSS Statistics 22 as well as Excel 2016 were used for statistical analyses. Baseline data are given as mean (SD) or percent as appropriate. Mean number of migraine days and days with pain medication are given as mean (SD) per cycle and for each week of the cycle for comparison with the HFI. To get more insight into the occurrence of prolonged attacks we also analysed the frequency of migraine attacks lasting longer than 24 h. Pain scores are presented as the mean score/migraine day for each week ofthe cycle. The number of severe migraine attacks (score 3) is reported for week 1 and as the mean of weeks 2–4 (weekly number/ 3). Diaries were started on the first day of pill use. To allow an easier comparison with the natural cycle, which starts with hormone withdrawal and bleeding we present our data starting with the HFI and presented this as days 1–7. Weeks two, three and four were the weeks with daily CHC use. Parameters of interest were reported as daily frequencies in percent and demonstrated as bar charts. For the HFI interval and for bleeding days − 3 to 5 we analysed in addition onset of migraine day and onset of bleeding. This time interval is slightly longer than that which is defined for MRM in the ICHD-3 classification in order not to miss an association in this special group. A similar procedure was used by MacGregor [11]. Daily frequency for onset of bleeding during the defined intervals was calculated. Comparisons of numeric data were performed using chi-square test or Wilcoxon test as appropriate. Relative risks for a migraine attack during pill use in comparison to the risk during the HFI were calculated with Cochran-Mantel-Haenszel test.

## Results

**Fig. 1:**
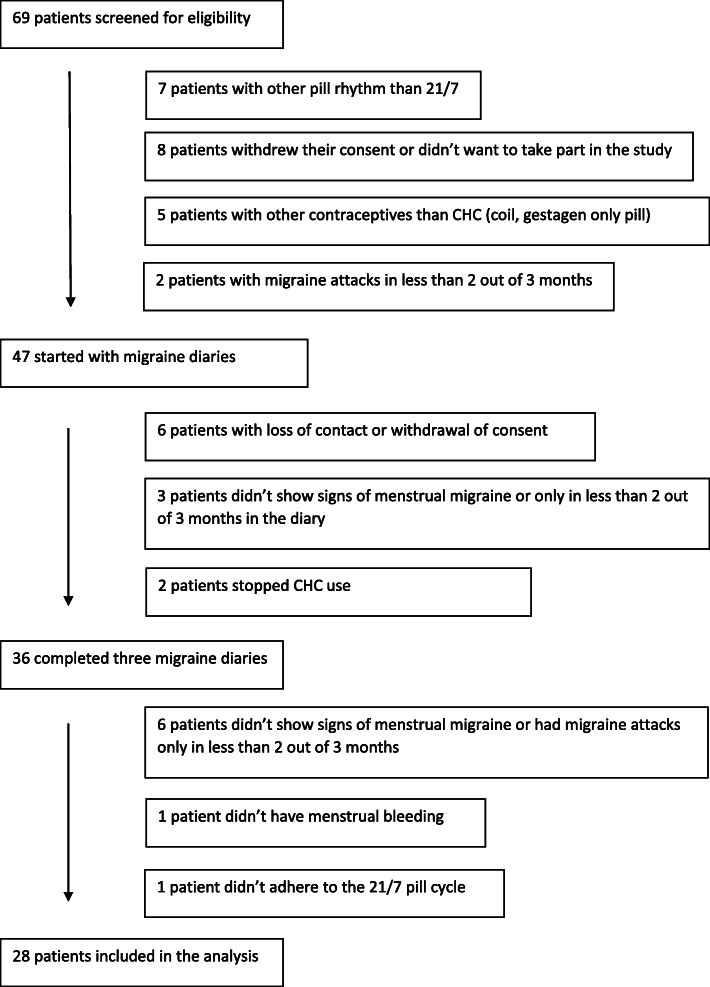
Flow chart of participants

Of 68 women screened, 47 were eligible to participate and 28 completed diaries over three cycles and continued to fulfil the inclusion criteria (Fig. 1). Altogether complete diaries of 84 CHC cycles and 84 HFI were analysed. Mean age of the 28 women was 28 (±5.3) years, height was 165 (±6) cm and weight was 59.4 (±12.9) kg. Mean age at the first migraine attack was 18.0 (±6.1) years, mean age at menarche was 13.1 ± 1.2 years. All participants used low-dose CHC with an ethinylestradiol dose ranging from 20 to 35 μg. Oral contraceptives were used by 22 women whereas six women used the vaginal ring. Most participants (96.5%) had never (68%) had aura, or had experienced aura only once (28.5%). Only 3.5% reported in the interview to have had an aura more than once. Many women (43%) stated that their migraine was not relieved by symptomatic medication.

Sixty-four percent of the participants experienced migraine during the HFI of all three cycles. One women did not experience withdrawal bleeding in one of the three HFI. Three of the 28 women were diagnosed with PMM and the remainder had MRM. The mean number of migraine days per 28-day cycle was 4.11 (±2.8), the mean number of migraines lasting more than 24 h (episodes) was 0.5 (±0.7) and the mean number of days with use of pain killers was 3.25 (±2.98). The mean pain score/migraine day was1.92 (range1–3).. Of all migraine attacks 104 were rated as severe (pain score 3) and 45% of the latter occurred during the HFI. The mean duration of migraine attacks lasting more than 24 h was 56.78 (±34.27) hours. Table 1 demonstrates the parameters of interest over the 28-days of the pill cycle and separate for each week [1–4]. During the HFI (presented as week 1) the number of migraine days, migraine attacks with a duration of more than 24 h, and consumed pain medication, were significantly higher in comparison with the weekly mean during hormone use. Also, the pain score/migraines day was significantly higher. The relative risk of having a migraine day on each day of the HFI in comparison with the mean risk during CHC use was significantly higher and fourfold for days 3–6 (Table 2).

**Table 1:** Migraine characteristics during the whole cycle and for each week of the cycle

Period	Migraine days	Days with pain medication	Mean Pain score/ migraine day (range 1–3)	Number of attacks with a duration of 24 h
Complete cycle (28 Days)	4.11 ± 2.81	3.25 ± 2.98	1.92	0.45 ± 0.72
Number week 1 (hormone-free interval)	2.18 ± 1.59	1.61 ± 1.34	1.98	0.29 ± 0.53
Number week 2 (pill use)	0.7 ± 0.98	0.55 ± 0.94	1.74	0.05 ± 0.21
Number week 3 (pill use)	0.55 ± 0.96	0.50 ± 0.9	1.86	0.06 ± 0.21
Number week 4 (pill use)	0.68 ± 1.19	0.61 ± 1.2	1.74	0.06 ± 0.24
*P*-value week 1 vs mean of week 2–4	< 0.0001	< 0.0001	< 0.03	< 0.0001

Pain score 0–3: 0 = no pain; 3 = severe pain

**Table 2:** Relative risk for the occurrence of migraine on each day of the hormone free interval compared to the pooled risk of day 8–28

	Relative risk	Confidence interval
**Day 1**	2.286	0.992–5.269
**Day 2**	2.286	0.992–5.269
**Day 3**	4.000	1.850–8.648
**Day 4**	4.714	2.210–10.055
**Day 5**	4.857	2.282–10.336
**Day 6**	4.000	1.850–8.648
**Day 7**	2.857	1.276–6.396

**Fig. 2:**
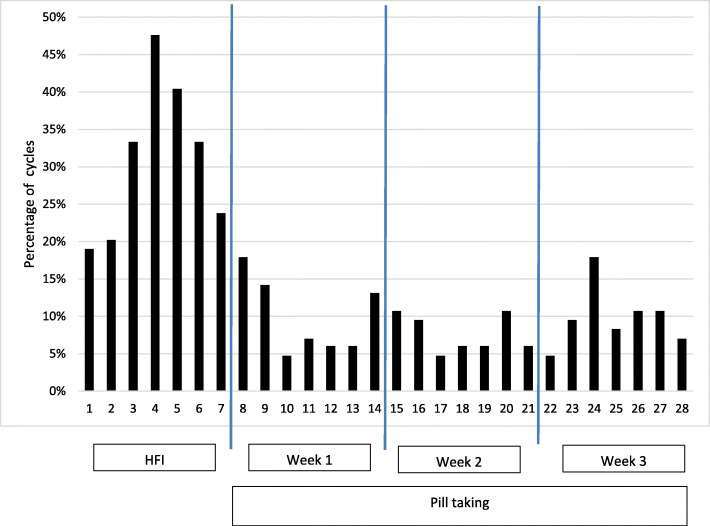
Migraine frequency for each day of the pill cycle

Figure 2 demonstrates the percentage of migraine days for each day of the pill cycle. Patients during the HFI were most likely to experience migrainous headache on day 4 (47.6%) followed by day 5 (40.4%) and days 3 and 6 with 33.3% each. The rate declined and was much lower one day after the first CHC intake (day 9).

**Fig. 3:**
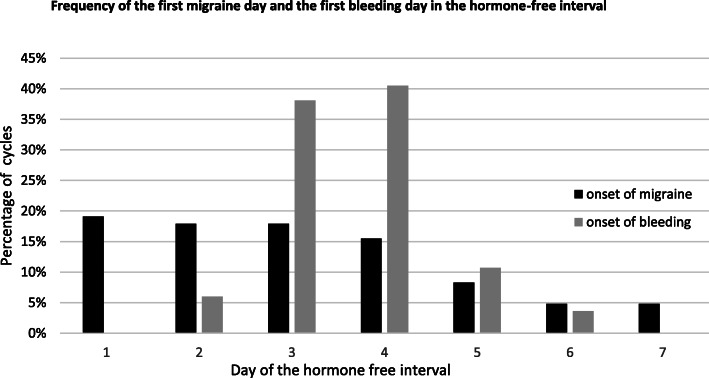
Frequency of the first migraine day and the first bleeding day in the hormone-free interval

**Fig. 4:**
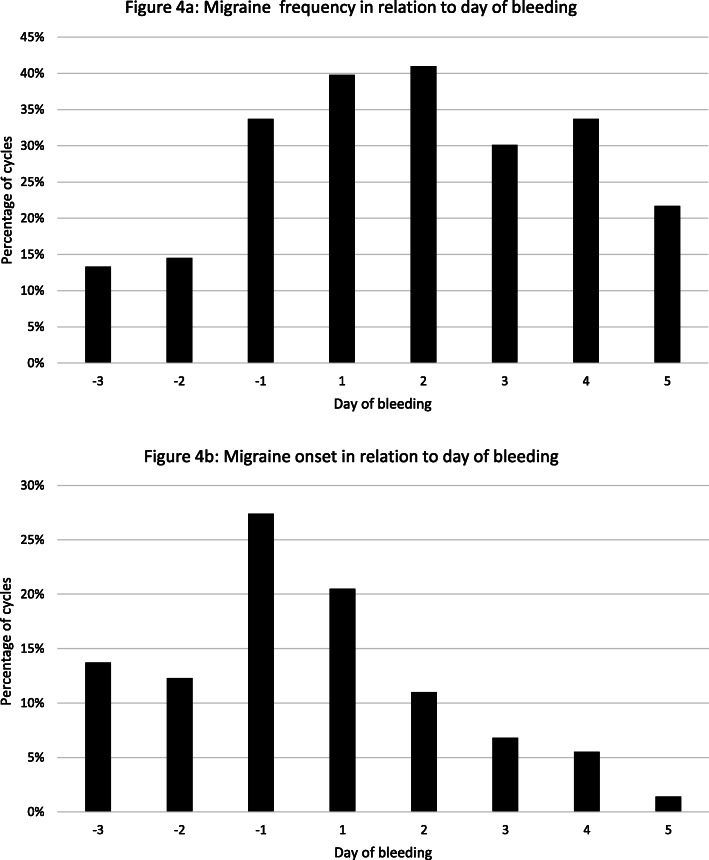
**a** /**b**: Migraine frequency and onset in relation to bleeding days day − 3 to day 5

The first migraine day during the HFI interval is of interest to plan the initiation of short-term prevention. Most migraines during the HFI started on days 1–4 (70.5%) (Fig. 3). In 58% of the cycles the first migraine day of the HFI occurred on days 3–7 and in 37.8% on days 4–7. The first bleeding day during the HFI occurred in around 80% of the cycles on days 3 and 4 (Fig. 3). Migraine in relation to bleeding mostly occurred at days − 1 to + 4 (Fig. 4a). We found that in 78% of the cycles the first migraine day occurred during bleeding days 1 ± 2, which is the period defined for the occurrence of menstrual migraine attacks. However, the typical start days of the menstrual attacks were day − 1 and the first day of bleeding (48%). Only around 25% of attacks started on bleeding days 2–5 (Fig. 4b). Migraine attacks with a duration of more than 24 h were observed in 45% of cycles, with 75% occurring during the HFI. The mean duration of these attacks was 56.8 h.

**Fig. 5:**
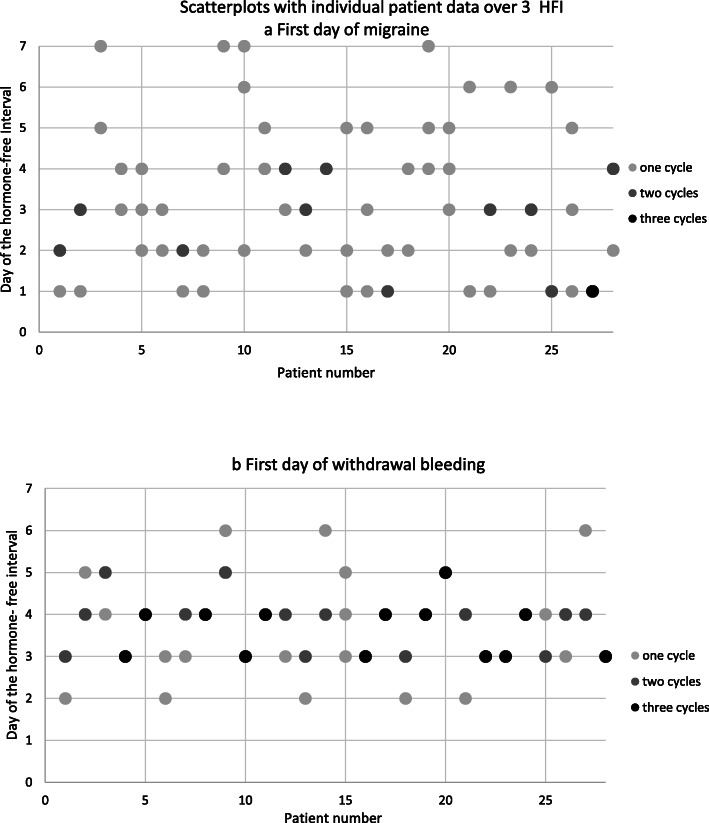
**a**/**b**: Scatterplots with individual patient data over 3 hormone-free intervals: a first migraine day and b: first bleeding day

The within-woman analysis showed that in 67% of the women the first migraine day varied by not more than two days, and in 42% varied only by one day from cycle to cycle (Fig. 5a). The first bleeding day was highly predictable with 42% of the participants always experiencing bleeding starting on the same day of the HFI, and in 82% it was either the same day or one day later (Fig. 5b).

## Discussion

In this prospective diary-based study in CHC users with menstrual migraine diagnosed in accordance with ICHD we found that most migraine days occurred during the HFI (Fig. 2). The days with the highest attack frequency (33% to 48%) were days 3–6 of the HFI. During these days the relative risk of a migraine attack was fourfold in comparison to days during CHC use (Table 1). The first migraine day occurred with a frequency between 15% and 19% on each of HFI days 1–4, very soon after hormone withdrawal. The frequency declined to 5–8% on HFI days 5–7 (Fig. 3). More relevant for the planning of treatment is our analyses of the first migraine day in relation to the first bleeding day, as we show, that this day is highly predictable in most CHC users. Almost 50% of attacks started on day − 1 and day 1 in relation to bleeding and 78% started on the day first bleeding day ±2 days. These results are in line with the timing defined for MRM in the ICHD. The incidence of migraine in CHC users was highest on days − 1 to day 4, which differs from natural cycles, in which the highest incidence of migraine is on days − 1 to 1 [25]. As for migraine attacks associated with the natural cycle, we found that menstrual attacks in CHC users were often rated as severe [11, 14, 18, 19, 24, 26]. Three quarter of all attacks were lasting longer than 24 h occurred during the HFI. Those long attacks occurred although women used their rescue medications as normal.

The distribution of migraine days over the HFI, the high predictability of the first bleeding day and the observation that migraine attacks start mostly on days − 1 to day + 4 in relation to the withdrawal bleed in CHC users confirm the results of our pilot study [23]. In line with our data Coffee et al. found that in 11 CHC users over 2 cycles the typical start of bleeding was at days 3 and 4 [27]. Headache scores in this trial were highest at days 3–7 of the HFI, which is similar to the days of the HFI with highest migraine prevalence in our study (days 3–6). The scores were still slightly elevated during the first two days of pill use, which is in line with our observation that migraine prevalence remains slightly elevated during the first two days of hormone restart, possibly due to the longer duration of attacks during the HFI. In the only trial of onset of migraine in relation to the HFI days 2–5 were identified as those with the highest prevalence of attacks in CHC users [7]. This contrasts to our data, which include day 1 of the HFI. A direct comparison of the data with ours is difficult as only women with PMM were included. Another trial comprised women with at least one migraine attack per month. Here the authors report the prevalence of migraine in CHC users in intervals of several days in relation to the withdrawal bleeding [24]. Migraine prevalence was highest during bleeding days 1–3 (HR 2.18, 95% CI 1.77–2.70), slightly elevated during the days − 2/− 1 (HR 1.32, 95% CI 1.01–1.72) and not significantly increased on other days of the cycle. The authors did not report onset of migraine days in relation to bleeding and migraine frequency for each day of the HFI. In an interesting recent retrospective cohort study oral contraceptive users more commonly reported menstrually-related worsening of headache in comparison to nonusers [28]. A limitation of this trial is that the authors included women with use of all types of oral contraception, including regimens without hormone-withdrawal. They did not report how many women used progestin-only pills or combined pills in a long-cycle regimen. Breakthrough bleeding in such continuous regimen is rather a consequence of hormone-induced changes of the endometrium than hormone-withdrawal.

Short-term prevention for menstrual attacks in women with PMM or MRM is recommended due to lack of efficacy of acute treatment for these attacks with fewer than 30% responding to acute triptan treatment [29, 30]. Most authors recommend to start prevention two days before the first expected headache day in the natural cycle or on day 21 of pill intake in CHC users and to continue for a period of 6–7 days [7, 16, 31, 32]. Adverse events associated with daily use of triptans and the fact that most migraineurs are likely to use additional triptans during CHC use, raise the question if such a long duration of prevention is necessary. Women with additional nonmenstrual attacks might risk medication overuse headache (MOH). In CHC users with the very high predictability of the first bleeding day an individually tailored shorter time frame for prevention might be the better option. Considering the high predictability of the first bleeding day, the observation that in 50% of cycles the first migraine day is day − 1 or + 1, and typically within the range of days 1 ± 2 we suggest to start prevention on bleeding day − 2 and continue for 3–4 days depending on the typical duration of the attacks. The exact interval of treatment could be individually adapted, if necessary based on diary data after two HFIs. The efficiacy of such an approach needs to be investigated in future trials. In one trial with a shortened HFI of 4 days use of frovatriptan reduced headaches but was unexpectedly associated with delayed headache after the four HFI days [33]. Preventing estrogen withdrawal with continuous/extended cycle is another management option that has been recommended for management of migraine during the HFI [34]. While a continuous combined CHC regimen might prevent migraine during the HFI, it would in contrast to use of the progestin-only pill with desogestrel, have no effect on migraine during hormone use. Morotti et al. found in a comparative trial with desogestrel and an extended cycle CHC regimen only a significant reduction in migraine days and pain medication in desogestrel users [35]. Preliminary studies indicate consistent, that switching to a progestin-only pill with desogestrel is the better option, as MRM attacks and nonmenstrual migraine attacks can be reduced. Furthermore use of this progestin is not associated with an increase in cardiovascular risk or stroke [35–40].

### Strengths and limitations

The strengths of the current study include the prospective design with daily conducted diaries, the confirmed diagnosis of migraine during an interview, the observation period of three complete cycles and the exclusion of CHC users with other than the conventional 21/7 CHC regimen. The within woman analyses of the first migraine day during the HFI is of high relevance and demonstrates how individual the reactions are with regard of migraine onset, despite the same CHC regimen. The study group consisted on women with mostly MRM and only few non-menstrual related migraine attacks. Therefore the comparison of the number of prolonged attacks between the HFI with weeks 2–4 has to be interpreted with caution. The strict inclusion criteria impeded recruitment and despite our interesting results, it must be acknowledged that this study was performed on a small number of subjects.

## Conclusion

Migraine attacks of CHC users are frequently severe and long lasting in the HFI. The highest prevalence of migraine was on days − 1 to 4 in relation to bleeding. The day of hormone-withdrawal migraine and the first bleeding day are highly predictable in CHC users. Migraine onset is mostly at day − 1 and 1 of the bleeding and on days 1–4 of the HFI. Further trials are necessary to investigate if this knowledge can be used to shorten and individualise the duration of prevention in CHC users. More research is needed to understand the pathophysiology of migraine during the HFI in CHC users with particular regard to estrogen withdrawal and/or uterine bleeding, and to optimize treatment.

## Data Availability

The datasets used for this study are available from the corresponding author on reasonable request.

## Abbreviations

CHC: Combined hormonal contraceptives
EWH: Estrogen withdrawal headache
HFI: Hormone-free interval
ICHD-3: The International Classification of Headache Disorders (ICHD-3)
IHS: International Headache Society
MRM: Menstrually-related migraine
MOH: Medication overuse headache
PMM: Pure menstrual migraine
